# Protocol for a systematic review of policies, programs or interventions designed to improve health and wellbeing of young people leaving the out-of-home care system

**DOI:** 10.1186/s13643-021-01792-5

**Published:** 2021-08-30

**Authors:** David J. A. Taylor, Aron Shlonsky, Bianca Albers, Sangita Chakraborty, Jane Lewis, Phillip Mendes, Geraldine Macdonald, Kevin Williams

**Affiliations:** 1 Department of Social Work, Monash University, 900 Dandenong Road, Caulfield East, VIC 3145 Australia; 2 Centre for Evidence and Implementation, 33 Lincoln Square South, Carlton, VIC 3053 Australia; 3 Institute for Implementation Science in Health Care, University of Zurich, Universitätstrasse 84, 8006 Zurich, Switzerland; 4 School for Policy Studies, University of Bristol, 8 Priory Rd, Bristol, BS8 1TZ UK; 5 The Fostering Network, 87 Blackfriars Road, London, SE1 8HA UK

**Keywords:** Systematic review, Transitions, Out-of-home care, Aftercare, Leaving Care, Ageing out

## Abstract

**Background:**

Relative to their counterparts in the general population, young people who leave, or transition out of, out-of-home (OOHC) arrangements commonly experience poorer outcomes across a range of indicators, including higher rates of homelessness, unemployment, reliance on public assistance, physical and mental health problems and contact with the criminal justice system. The age at which young people transition from OOHC varies between and within some countries, but for most, formal support ceases between the ages of 18 and 21.

Programs designed to support transitions are generally available to young people toward the end of their OOHC placement, although some can extend beyond. They often encourage the development of skills required for continued engagement in education, obtaining employment, maintaining housing and general life skills. Little is known about the effectiveness of these programs or of extended care policies that raise the age at which support remains available to young people after leaving OOHC. This systematic review will seek to identify programs and/or interventions that improve outcomes for youth transitioning from the OOHC system into adult living arrangements.

**Methods:**

This review will identify programs, interventions and policies that seek to improve health and wellbeing of this population that have been tested using robust controlled methods. Primary outcomes of interest are homelessness, health, education, employment, exposure to violence and risky behaviour. Secondary outcomes are relationships and life skills. We will search, from January 1990 onwards, MEDLINE, EMBASE, PsycINFO, ERIC, CINAHL, Cochrane CENTRAL, SocINDEX, Sociological Abstracts, Social Services Abstracts, NHS Economic Evaluation Database and Health Technology Assessment. Grey literature will be identified through searching websites and databases, e.g. clearing houses, government agencies and organisations known to be undertaking or consolidating research on this topic area. Two reviewers will independently screen all title and abstracts and full text articles with conflicts to be resolved by a third reviewer. Data extraction will be undertaken by pairs of review authors, with one reviewer checking the results of the other. If more than one study with suitable data can be identified, we plan to undertake both fixed-effects and random-effects meta-analyses and intend to present the random-effects result if there is no indication of funnel plot asymmetry. Risk of bias will be assessed using tools appropriate to the study methodology. Quality of evidence across studies will be assessed using the Grading of Recommendations, Assessment, Development and Evaluation (GRADE) methodology.

**Discussion:**

Previous reviews were unable to identify any programs or interventions, backed by methodologically rigorous research, that improve outcomes for this population. This review seeks to update this previous work, taking into account changes in the provision of extended care, which is now available in some jurisdictions.

**Systematic review registration:**

PROSPERO CRD42020146999

**Supplementary Information:**

The online version contains supplementary material available at 10.1186/s13643-021-01792-5.

## Background

Youth who experience abuse and neglect by their parents or carers can be placed in out-of-home care (OOHC) in jurisdictions where such formal systems exist. OOHC takes three major forms: foster care—where care services are provided by individuals not necessarily known to the recipient, kinship care—where those providing care are connected to the recipient through blood or kin ties, and residential care—where care is provided in an institutional setting. Youth can experience one or more of these care types whilst in OOHC. Whilst the three forms of OOHC are quite different, they also have similarities: children in homes were often the victims of childhood trauma [1, 2]; minimal standards of care are required; and financial support, if provided, ceases when youth reach a certain age [3].

OOHC is a policy area of considerable contemporary cross-national interest [4]. The latest available figures for England show that there were 75,420 children in care in 2018. In the same year, 10,460 young people aged 17–18 left care who had spent at least 13 weeks in care between the age of 14 and 16 [5]. In Australia, there are approximately 45,000 children in care, with 3300 of them aged between 15 and 17 leaving care arrangements in 2018-2019 [6]. Comparable figures for the USA show that there were 122,000 children aged between 13 and 20 in foster care on 30 September 2018. Of these, approximately 20,000 left care in FY2017, because they reached the legal age of adulthood in their state [7]. Similar statistics for European countries, representing a broad range of different welfare state regimes and thus approaches to OOHC, are more difficult to retrieve. According to a recent report, 161,233 young people aged 0–17 years were in OOHC in Germany in 2013, and additional 28,181 were characterised as “care leavers”, i.e. aged 18–26 years [8]. In Denmark, 13,532 children were placed in OOHC by December 31, 2020. Of these, 3,928 were 15–17 years old [9].

Young people who leave or transition out of OOHC arrangements commonly experience poorer outcomes compared to the general population across a range of indicators, including higher rates of homelessness, unemployment, reliance on public assistance, physical and mental health problems and contact with the criminal justice system [10–16]. These poorer outcomes may be due to pre-existing mental health problems and other challenges arising from their experiences pre-care or whilst in care [17, 18]. They may also be due to insufficient knowledge or life skills or be related to the fact that they must fend for themselves at an earlier age than their peers who can rely on their birth families for personal and material support [17].

The age at which young people transition from OOHC varies between and within some countries—for most, formal support ceases between the ages of 18 and 21 [19]. The type and mode of support to care leavers varies between jurisdictions, but can involve formal life skills training programs, personal adviser or key worker support, mentoring or peer support programs, cash assistance and housing support [17, 20]. Young people transitioning from care are often ill-equipped for independent living, and the type and amount of support they receive is insufficient to prevent adverse outcomes [21, 22]. Considering this, in some jurisdictions, one policy response has been to raise the age at which young people can transition, thereby extending the support available [4, 23, 24].

Transition support programs are generally available to young people toward the end of their care placement, although some extend beyond. They often encourage the development of skills required for continued engagement in education, obtaining employment, maintaining housing and general life skills [17, 20].

Fifteen years ago, Donkoh et al. conducted the first methodologically rigorous systematic review of independent living programs for young people leaving out-of-home care and were unable to find any studies that met their inclusion criteria [17]. In the intervening period, a number of reviews have explored various aspects of policies, programs or interventions for youth transitioning from care but have suffered from a range of weaknesses in either methodology or scope.

Some reviews have limited their scope, either to particular geographies [20], or to interventions delivered whilst youth were in care [17, 25], or to independent living programs [17, 26, 27]. Some focused on a narrow range of outcomes [27, 28]. Others have methodological weaknesses, such as not conducting a systematic search [22], not applying a methodological filter or addressing the risk of bias of included studies [25, 29], or they failed to critically appraise the effectiveness of the policy or practice interventions included [26, 27, 30–32]. This review seeks to update and build upon this previous work by additionally investigating the effectiveness of policies that provide the option to extend out-of-home care beyond 18 years old, thereby increasing the age at which young people transition.

The aim of this systematic review is to assess the effectiveness of programs and/or interventions designed to improve outcomes for youth transitioning from the out-of-home care system into adult living arrangements. The proposed systematic review question is:

What programs, interventions or services are effective at improving health and psychosocial outcomes for young people leaving the out-of-home care system?

## Methods

The systematic review has been registered with the International Prospective Register of Systematic Reviews (PROSPERO, http://www.crd.york.ac.uk/PROSPERO, registration number: CRD42020146999).

The present protocol is being reported in accordance with the reporting guidance provided in the Preferred Reporting Items for Systematic Reviews and Meta-Analyses Protocol (PRISMA-P) [33] (see Additional file 1). The final review will be reported in accordance with the updated PRISMA 2020 statement [34] .

### Criteria for considering studies for this review

Studies will be selected according to the following criteria: population, intervention, comparator, outcome and study design.

### Population

Youth aged between 16 and 25 who are:

- Not living with their birth parents/family; AND
- Are in foster care/out-of-home care/public care/looked after (UK)/state care/government care; AND
- Have been placed in care due to concerns related to child maltreatment, neglect or risk of child maltreatment, relinquishment, or lack of provision of support; AND
- Who are transitioning from care into adult living arrangements.

### Intervention

Policies, programs or interventions that:

- Provide support and/or assistance to help youth prior to leaving care and/or as they transition and/or after they leave care;
- Are delivered in the community;
- Support young people transition from their country’s statutory out-of-home care systems into adult living.

### Comparator

The following comparisons will be included: intervention/services as usual (i.e. what an individual would have received in the absence of the intervention), another intervention (i.e. another policy, program or intervention for young people leaving out-of-home care), no intervention or wait-list control (i.e. individuals waiting to be included in the intervention).

### Outcome(s)

Outcomes of interest include the following, which must be measured at least three months following the age at which eligibility for standard out-of-home care terminates in the jurisdiction in which the study took place. Outcomes will be considered if they were obtained from linked administrative data sources (i.e. employment, health or other records), validated measures (e.g. conflict tactics scale) and non-validated measures (e.g. self-reported homelessness) administered by interview or survey.

### Primary

- *Homelessness*—we will include any homelessness/housing-related outcomes, including any measurement that allows us to determine whether or not an individual has or does not have a permanent place to live;
- *Health*—we will include any health-related outcomes or measures of service usage, including but not limited to emergency department presentations, hospitalisations and any measure of mental health conditions or symptoms;
- *Education*—we will include any education-related outcomes, including but not limited to measurement of high school or equivalent completion, high school grades, enrolment in or attainment of a trade/vocational qualification and enrolment in or attainment of a university qualification;
- *Employment*—we will include any employment-related outcomes, including but not limited to measurement of whether an individual is employed, their wages or utilises unemployment benefits;
- *Exposure to violence (as either victim or perpetrator)*—we will include any exposure to violence-related outcomes, including but not limited to measurement of crime perpetration (i.e. whether or not an individual has been arrested, convicted, or spent time in a locked setting, e.g. jail/prison) or crime victimisation (i.e. where the individual was the victim);
- *Risky behaviour*—we will include any risky behaviour-related outcomes, including but not limited to measurement of illicit drug use, alcohol abuse, risky sexual behaviour, positive sexually transmitted infection tests and either the onset or delay of teen pregnancy.

### Secondary

- *Relationships*—we will include any relationships-related outcomes, including but not limited to measurement of whether an individual has attained or maintained supportive relationships with others, including mentors, peer mentors or supportive peers;
- *Life skills*—we will include any life skills-related outcomes, including but not limited to measurement of the attainment of competencies required for independent living; these include, but are not limited to, learning how to budget, attain essential services and perform essential household tasks.

### Study design(s)

The following experimental and quasi-experimental study designs will be included: randomised controlled trials (RCTs) including individual RCTs, cluster RCTs, Step-Wedge designs with random time allocation and non-equivalent control group designs using parallel cohorts that adjust for baseline equivalence; difference-in-difference estimation, synthetic control group methods, studies based on covariate matching, propensity score-based methods, doubly robust methods, regression adjustment, regression discontinuity designs and instrumental variable estimation; and economic evaluation methodologies including cost-benefit analysis, cost-utility analysis, cost-effectiveness analysis and cost-analysis.

Economic evaluations and qualitative studies will be included if they are conducted as part of a qualifying study and will be used only to inform or deepen our understanding of the quantitative findings.

### Information sources and search strategy

The following databases will be searched for studies published from January 1990 onward using strategies included in (Additional file 2):

- Cochrane CENTRAL
- CINAHL
- ERIC
- PsycINFO
- MEDLINE
- EMBASE
- Sociological Abstracts
- Social Services Abstracts
- SocINDEX
- NHS Economic Evaluation Database
- Health Technology Assessment

### Supplementary searches

Websites and databases from clearinghouses, government agencies and organisations known to be undertaking or consolidating research in this area from the USA, Europe, Australia, Canada and New Zealand will be examined for unpublished material, including but not limited to:

- Social Care Online (SCIE)
- International Research Network on Transitions to Adulthood from Care
- Analysis and Policy Observatory
- Australian Institute of Family Studies
- Chapin Hall at the University of Chicago
- California Evidence-Based Clearinghouse for Child Welfare
- Washington State Institute for Public Policy

In addition to the organisations listed above, we will search the websites of organisations affiliated with authors or included studies.

The reference lists of previous systematic reviews identified in the search process will be reviewed alongside those reviews already known to the authors. References of included studies will also be screened for eligibility. Additionally, authors of included studies will be contacted by email to ascertain if they are aware of any additional unpublished literature that may meet the inclusion criteria for this review.

### Language of publication

No restrictions will be placed on the language of publication.

### Data collection and management

Citations identified from the search strategy will be imported into the online systematic review application Covidence [35] for screening. Following PRISMA guidelines, a flowchart will be provided demonstrating the inputs and results of each stage of the review process.

### Study selection

Title and abstracts will be reviewed independently by two review authors, with a third reviewer resolving conflicts when they arise. Two review authors will independently read the full-text versions of all potentially eligible studies that they have selected, and a third reviewer will resolve conflicts when necessary. The eligibility criteria are included in Table 1.

**Table 1:** Inclusion and exclusion criteria by PICO domain

PICO	Inclusion criteria	Exclusion criteria
*Study design*	• Randomised controlled trials (RCT) including: ▪ Individual RCTs ▪ Cluster RCTs • Step-Wedge designs with random time allocation • Non-equivalent control group designs using parallel cohorts that adjust for baseline equivalence • Difference-in-difference estimation • Interrupted time-series • Synthetic control group methods • Studies based on: ▪ Covariate matching ▪ Propensity score based methods, ▪ Doubly robust methods ▪ Regression adjustment ▪ Regression discontinuity designs, and ▪ Instrumental variable estimation. Qualitative studies and economic evaluations will be included if they are conducted as part of a qualifying study and will be used only to generate hypotheses, inform us about the interventions and populations and inform or deepen our understanding of the quantitative findings.	• Non-primary studies, including: ▪ Literature reviews ▪ Systematic reviews ▪ Meta-analysis • Studies without a valid counterfactual, including designs that do not include a parallel cohort that establish or adjust for baseline equivalence, including: ▪ Single group pre-post designs ▪ Control group designs without matching in time and establishing baseline equivalence ▪ Cross-sectional designs ▪ Non-controlled observational (cohort) designs ▪ Case-control designs ▪ Case studies/series ▪ Surveys Qualitative designs and economic evaluations not undertaken in the context of an included quantitative study.
*Population*	Youth aged between 16 and 25 Youth in OOHC for reasons of child maltreatment, neglect or risk of child maltreatment, relinquishment or lack of provision of support. OOHC settings include: • Foster care • Guardianship • Kinship care • Group care • Residential care • Congregate care	Youth in OOHC settings for reasons other than child maltreatment, neglect, risk of child maltreatment, relinquishment or lack of provision of support. Youth who are not in OOHC. Youth who are currently incarcerated, including in youth justice settings. Youth aged less than 16 and greater than 25.
*Intervention*	Policies, programs or interventions delivered in the home or community.	Policies, programs or interventions delivered in other settings, for example: custodial settings. Policies, programs or interventions where the focus is not on youth transitioning from out-of-home care.
*Comparison*	Treatment as usual, another intervention, no intervention, or wait-list control.	Studies using other comparators.
*Outcome*	Primary outcomes: • Homelessness • Health • Education • Employment • Exposure to violence (as either victim or perpetrator) • Risky behaviour Secondary outcomes: • Relationships • Life skills	Studies looking at other outcomes.
*Setting*	Countries where a statutory care system for child maltreatment exists.	Countries where a statutory care system for child maltreatment does not exist.

### Data extraction

Data extraction will be undertaken by pairs of review authors, with one reviewer checking the results of the other. Data will be extracted into an online form developed for this review. The following information will be obtained: study design, study outcomes, sample size, location, study timeframe, study population, population demographics, intervention details, effect sizes, implementation outcomes as well as information required to conduct risk of bias assessments. Authors of included studies will be contacted by email to request any required information that is unavailable in print.

### Data analysis

We will synthesise data around each of our primary outcome domains (as defined in the “Criteria for considering studies for this review” section) as well as considering author affiliation, funding sources, conflicts of interest, individual versus group delivery and setting. If sufficient studies are identified, subgroup analysis will be undertaken based upon the following characteristics: (a) study design: randomised control trials vs. non-randomised control trials, (b) age at which statutory out-of-home care support ceases: 18 vs. greater than 18 and (c) gender.

For binary outcomes, we will calculate a risk ratio and a 95% confidence interval. For continuous data, if a consistent outcome measure is used, we will calculate the mean difference. Where different measures are used to examine primary outcomes, we will calculate the standardised mean difference if possible. Where data from the same outcome are reported in different studies as dichotomous or continuous data, we will transform these (if appropriate) to enable pooled estimates of effect.

If effect sizes are missing or displayed in ways that do not allow us to determine a pooled estimate, we will use the Campbell Collaboration effect size calculator [36]. If we are not able to calculate an effect size in this manner, we will contact the authors and seek to obtain the information required for us to do so.

In accordance with the Cochrane Handbook for Systematic Reviews of Interventions, we will try to maximise the likelihood of being able to quantitatively synthesise primary and secondary outcomes across studies using a meta-analysis [37]. If more than one study with suitable data can be identified, we intend to perform a meta-analysis if the population, intervention, comparison and outcomes are similar enough to be reasonably combined or can be standardised for comparison. To make this judgement, we will group included studies by outcome domain and study design, whilst considering the intervention type, population, comparator, outcome measure and timing of outcome measurement.

We plan to undertake both fixed-effects and random-effects meta-analyses and intend to present the random-effects result if there is no indication of funnel plot asymmetry. These models will allow us to estimate the pooled effect size and its 95% confidence interval for each outcome. For both approaches, we will perform a meta-analysis using the longest follow-up period possible for each outcome.

Data from randomised and non-randomised trial designs will not be pooled. We will also not pool data from non-randomised studies of different study designs. We will seek to undertake sensitivity analysis, if appropriate based on decisions made during the review process.

For each outcome, we will explore heterogeneity by preparing box plots, forest plots and examining the *I*^2^ statistic. Evidence of heterogeneity—where the *p* value < 0.1 and *I*^2^ statistic is greater than 75%—will be highlighted in the reporting of that outcome. Thresholds of low, medium and high heterogeneity will be assigned to *I*^2^ values of 25%, 50% and 75% [38]. This analysis will be conducted using the R Project for Statistical Computing [39].

If the conditions for conducting a meta-analysis are not met, we will describe and synthesise study findings narratively in line with guidance from the SWiM reporting guideline [40]. The data from each included study (e.g. study characteristics, context, exposures, outcomes and findings) will be used to build summary of findings/evidence tables including an overall description of included studies. Studies will be grouped, and results synthesised by outcome domain, stratified by study design, intervention type and population. We will use descriptive measures (median effect size and confidence interval) to describe their effects. We will use Cohen’s benchmarks to assist in the interpretation of the magnitude of both dichotomous and continuous effect sizes where: small (SMD = 0.2), medium (SMD = 0.5) and large (SMD = 0.8) [41].

For studies included in a meta-analysis, publication bias will be assessed by visually assessing funnel plot asymmetry. For continuous outcomes measured as mean differences Egger’s regression test will be used [37].

### Evidence assessment

Risk of bias assessments will be used to explore heterogeneity, to inform decisions regarding the suitability of conducting a meta-analysis and to assess the strength of inferences supporting Grading of Recommendations, Assessment, Development and Evaluation (GRADE) recommendations. Risk of bias of included randomised controlled trials will be assessed using the Cochrane Risk of Bias 2 (RoB 2) tool [42]. Non-randomised studies will be assessed using the ROBINS-I tool [43]. The quality of any economic evaluations will be assessed using guidelines suggested by the Cochrane and Campbell Economics Methods Group [44]. The quality of evidence across outcomes that can be synthesised in a meta-analysis will be assessed as either very low, low, moderate or high using the GRADE methodology [45].

## Discussion

Reflecting its place as an emergent policy issue, the volume of research published on care leavers has increased substantially in the last 20 years, but this literature has not been the subject of high-quality synthesis. Therefore, it is an appropriate time for a systematic review to inform ongoing policy and practice discussions about how best to support young people transitioning from care and to shape future research.

This review has a number of advantages over previously published work on this topic, including (a) a systematic search of a wide range of databases with no language restrictions, (b) the use of inclusion criteria that prioritise studies with high-quality methodologies, (c) the selection of outcomes that reflect health and psychosocial wellbeing, (d) the inclusion of policy alongside individual or group-level interventions and (e) the use of the GRADE approach to assess the quality of evidence, report results and support knowledge translation.

In conducting this review, we also expect to encounter limitations. Including studies from different countries and settings, representing different social welfare regimes and policies as well as different social care systems may make it difficult to combine findings from studies. To address this concern, we will take these and other contextual factors into account in analysing, synthesising and reporting findings. Additionally, the inclusion of studies in our review that use a wide range of outcome measurements to assess similar constructs will likely limit the scope of any meta-analyses that we can undertake. As highlighted above, if this is the case, we will report findings without conducting meta-analyses using the SWiM guidelines.

The results of this review will have the potential to inform policy discussions in this area in a number of jurisdictions, as they evaluate the merits of providing different modes of transitions support, raising the age at which OOHC can be provided to young people or a combination of both.

The results of this review will also provide guidance to organisational leaders and other sector decision makers involved in the design and delivery of out-of-home care, transitions and independent living programs by identifying potentially promising interventions and/or key aspects important to take into account when supporting young people transitioning out of OOHC.

However, it will be important to interpret findings from this review with great caution. Previous international studies of out-of-home care systems and policies have emphasised that studies conducted in different welfare state regimes may represent different ways of defining and conceptualising out-of-home care, its key target population and key aspects of the care experience [4, 16, 46]. In synthesising these as part of this review, detailed information on the context dependence of findings may therefore get lost, highlighting the need for a locally driven translation process of results and their potential implications for policy and practice in specific locations.

The results of this review will be disseminated though publication in a report by the funder, conference presentations and in peer reviewed publications. Any future amendments to this protocol in the course of the review will be updated in PROSPERO and reported in the published review.

## Supplementary Information

**Additional file 1.** PRISMA-P 2015 Checklist.

**Additional file 2.** Search strategy.

## Data Availability

Not applicable.

## Abbreviations

GRADE: Grading of Recommendations, Assessment, Development and Evaluation
OOHC: Out-of-home care
QED: Quasi-experimental design
RCT: Randomised control trial
SWiM: Synthesis without meta-analysis
