# Supplementary material for: Protocol for a systematic review of policies, programs or interventions designed to improve health and wellbeing of young people leaving the out-of-home care system
Source: Syst Rev. 2021 Aug 30;10:240. doi: 10.1186/s13643-021-01792-5 (PMC8404288; doi:10.1186/s13643-021-01792-5)
Supplement: Supplementary file 2 — Additional file 2. Search strategy. [file 13643_2021_1792_MOESM2_ESM.pdf]

Search strategy

Cochrane Controlled Register of Trials via Ovid

1. child welfare/ or foster home care/
2. (foster adj2 (youth or child* or care)).ti.
3. (foster adj2 (youth or child* or care)).ab
4. Independent Living/
5. independent living.ti.
6. independent living.ab
7. Self Care/
8. (extend* adj2 (care or foster* or out of home care or OOHC or looked after)).mp.
9. (leav* adj2 (care or foster* or out of home care or OOHC or looked after)).mp.
10. (transit* adj2 (care or foster* or out of home care or OOHC or looked after)).mp.
11. (ag* out adj2 (care or foster* or out of home care or OOHC or looked after)).mp.
12. (emancipat* adj2 (care or foster* or out of home care or OOHC or looked after)).mp
13. 1 or 2 or 3
14. 4 or 5 or 6 or 7 or 8 or 9 or 10 or 11 or 12
15. 13 and 14
16. (RCT or Trial* or randomi* or random* allocat* or random* assign* or (control* adj1 Intervention*) or (treatment* adj1 control*) or evaluat* study or control group* or control condition* or comparison group* or comparison condition* or time series or (before adj1 after) or pre post or longitudinal or repeated measures or effect size* or comparative effective* or experiment* or pre-experiment* or difference in difference* or instrumental variable* or Propensity score or (control* adj1 treat*) or wait* list or quasi ex* or quasiexperiment* or matched control or matched comparison).ti.
17. (RCT or Trial* or randomi* or random* allocat* or random* assign* or (control* adj1 Intervention*) or (treatment* adj1 control*) or evaluat* study or control group* or control condition* or comparison group* or comparison condition* or time series or (before adj1 after) or pre post or longitudinal or repeated measures or effect size* or comparative effective* or experiment* or pre-experiment* or difference in difference* or instrumental variable* or Propensity score or (control* adj1 treat*) or wait* list or quasi ex* or quasiexperiment* or matched control or matched comparison).ab
18. Clinical Trial or Empirical Study or Experimental Replication or Followup Study or Longitudinal Study or Prospective Study or Retrospective Study or Quantitative Study or Treatment Outcome or Field Study or Mathematical Modeling).mp.
19. 16 or 17 or 18
20. 15 and 19

CINAHL via EBSCO

1. (MM “Foster Home Care”) OR (MH “Foster Parents”) OR (MH “Child, Foster”)
2. (MH “Child Welfare+”)
3. TI foster n2 child* OR TI foster n2 youth OR TI foster n2 parent* OR TI foster n2 care* OR TI foster n2 home
4. AB foster n2 child* OR AB foster n2 youth OR AB foster n2 parent* OR AB foster n2 care* OR AB foster n2 home
5. (TI (extend* n2 care or foster*)) OR (AB (extend* n2 care or foster*))
6. (TI (leav* n2 care or foster*)) OR (AB (leav* n2 care or foster*))
7. (TI (transit* n2 care or foster*)) OR (AB (transit* n2 care or foster*))
8. (TI (ag* out n2 care or foster*)) OR (AB (ag* out n2 care or foster*))
9. 1 OR 2 OR 3 OR 4
10. 5 OR 6 or 7 or 8

11. 9 AND 10
12. (MH "Randomized Controlled Trials") OR (MH "Clinical Trials")
13. (MH "Evaluation" OR ("MH Program Evaluation")
14. TI "Randomized Controlled Trials" OR TI "Clinical Trials"
15. (MH "Quasi-Experimental Studies+")
16. (MH "Quasi-Experimental Studies") OR (MH "Nonequivalent Control Group") OR (MH "Time Series") OR (MH "Repeated Measures") OR (MH "Retrospective Design") OR (MH "Time and Motion Studies")
17. (quasi-experiment* OR quasiexperiment* OR "propensity score*" OR "control* group*" OR "control condition*" OR "treatment group*" OR "comparison group*" OR "wait-list*" OR "waiting list*" OR "intervention group*" OR "experimental group*" OR "matched control*" OR "matched groups" OR "matched comparison" OR "experimental trial" OR "experimental design" OR "experimental method*" OR "experimental stud*" OR "experimental evaluation" OR "experimental test*" OR "experimental assessment" OR "comparison sample" OR "propensity matched" OR "control sample" OR "control subject*" OR "intervention sample" OR OR "no treatment group" OR "nontreatment control" OR "pseudo experimental" OR "pseudo randomi?ed" OR "quasi-RCT" OR "quasi-randomi?ed" OR "compared with control*" OR "compared to control*" OR "compared to a control*" OR "non-randomi?ed controlled stud*" OR "nonrandom* assign*")
18. 12 or 13 or 14 or 15 or 16 or 17
19. 11 and 18

ERIC via Proquest

S1. MAINSUBJECT.EXACT("Child Safety") OR MAINSUBJECT.EXACT("Child Welfare") OR MAINSUBJECT.EXACT("Foster Care")
S2. ti(foster N/2 child*) OR ti(foster N/2 parent*) OR ti(foster N/2 care*) OR ti(foster N/2 home*)) OR (ab(foster N/2 child*) OR ab(foster N/2 parent*) OR ab(foster N/2 care*) OR ab(foster N/2 home)
S3. MAINSUBJECT.EXACT("Independent living") OR MAIN SUBJECT.EXACT("Daily living") OR ((extend* NEAR/2 (care OR foster*)) OR (leav* NEAR/2 (care OR foster*)) OR (transit* NEAR/2 (care OR foster*)) OR (ag* out NEAR/2 (care OR foster*))) OR su("Transitional programs")
S4. S1 OR S2
S5. S3 AND S4
S6. RCT OR Trial* OR randomi* OR "random* allocat*" OR "random* assign*" OR (control* n/1 intervention*) OR (treatment* n/1 control*) OR "evaluat* study" OR "control group*" OR "control condition*" OR "comparison group*" OR "comparison condition*" OR "time series" OR "before after") OR ("pre post" OR longitudinal OR "repeated measures" OR "effect size*" OR "comparative effective*" OR experiment* OR pre-experiment* OR "difference?in?difference*" OR "instrumental variable*" OR "propensity score*" OR (control* n/1 treat*) OR "wait* list" OR "quasi ex*" or quasiexperiment* OR "matched control" OR "matched comparison"
S7. (MAINSUBJECT.EXACT("Control Groups") OR MAINSUBJECT.EXACT("Matched Groups") OR MAINSUBJECT.EXACT("Quasiexperimental Design") OR MAINSUBJECT.EXACT("Randomized Controlled Trials") OR MAINSUBJECT.EXACT("Program Evaluation") OR MAINSUBJECT.EXACT("Outcomes of Treatment") OR MAINSUBJECT.EXACT("Medical Care Evaluation") OR MAINSUBJECT.EXACT("Replication (Evaluation)") OR MAINSUBJECT.EXACT("Evaluation Research") OR MAINSUBJECT.EXACT("Scientific Research") OR MAINSUBJECT.EXACT("Therapy") OR MAINSUBJECT.EXACT("Cost Effectiveness") OR MAINSUBJECT.EXACT("Medical Evaluation") OR MAINSUBJECT.EXACT("Program Effectiveness") OR MAINSUBJECT.EXACT("Outcome Measures") OR MAINSUBJECT.EXACT("Experimental Groups") OR MAINSUBJECT.EXACT("Experimental Programs") OR MAINSUBJECT.EXACT("Data Analysis") OR MAINSUBJECT.EXACT("Comparative Analysis") OR MAINSUBJECT.EXACT("Intervention"))

S8. S6 OR S7
S9. S5 AND S8

PsycINFO via Ovid

1. foster care/ or child welfare/ or foster children/ or foster parents/ or protective services/
2. (foster adj2 (youth or child* or care)).ti.
3. (foster adj2 (youth or child* or care)).ab.
4. independent living programs/
5. independent living.ti.
6. independent living.ab.
7. self-care skills/
8. self-determination/
9. (extend* adj2 (care or foster* or out of home care or OOHC or looked after)).mp.
10. (leav* adj2 (care or foster* or out of home care or OOHC or looked after)).mp.
11. (transit* adj2 (care or foster* or out of home care or OOHC or looked after)).mp.
12. (ag* out adj2 (care or foster* or out of home care or OOHC or looked after)).mp.
13. (emancipat* adj2 (care or foster* or out of home care or OOHC or looked after)).mp
14. 1 or 2 or 3
15. 4 or 5 or 6 or 7 or 8 or 9 or 10 or 11 or 12 or 13
16. 14 and 15
17. (Clinical Trial or Empirical Study or Experimental Replication or Followup Study or Longitudinal Study or Prospective Study or Retrospective Study or Quantitative Study or Treatment Outcome or Field Study or Mathematical Modeling).md
18. (RCT or Trial* or randomi* or random* allocat* or random* assign* or (control* adj1 Intervention*) or (treatment* adj1 control*) or evaluat* study or control group* or control condition* or comparison group* or comparison condition* or time series or (before adj1 after) or pre post or longitudinal or repeated measures or effect size* or comparative effective* or experiment* or pre-experiment* or difference in difference* or instrumental variable* or propensity score or (control* adj1 treat*) or wait* list or quasi ex* or quasiexperiment* or matched control or matched comparison).ti.
19. (RCT or Trial* or randomi* or random* allocat* or random* assign* or (control* adj1 Intervention*) or (treatment* adj1 control*) or evaluat* study or control group* or control condition* or comparison group* or comparison condition* or time series or (before adj1 after) or pre post or longitudinal or repeated measures or effect size* or comparative effective* or experiment* or pre-experiment* or difference in difference* or instrumental variable* or propensity score or (control* adj1 treat*) or wait* list or quasi ex* or quasiexperiment* or matched control or matched comparison).ab.
20. 17 or 18 or 19
21. 16 and 20

MEDLINE via Ovid

1. exp Foster Home Care/or exp Child Welfare/ or exp Child, Foster/ or foster care.mp
2. child protective services.mp or Child protective services/
3. (foster adj2 (youth or child* or care)).ti
4. (foster adj2 (youth or child* or care)).ab
5. exp Independent living/ or exp self care/ or exp self-neglect/ or exp social participation
6. independent living.ti
7. independent living.ab
8. (extend* adj2 (care or foster* or out of home care or OOHC or looked after)).mp
9. (leav* adj2 (care or foster* or out of home care or OOHC or looked after)).mp.
10. (transit* adj2 (care or foster* or out of home care or OOHC or looked after)).mp.
11. (ag* out adj2 (care or foster* or out of home care or OOHC or looked after)).mp
12. (emancipat* adj2 (care or foster* or out of home care or OOHC or looked after)).mp
13. 1 or 2 or 3 or 4
14. 5 or 6 or 7 or 8 or 9 or 10 or 11 or 12

15. 13 and 14
16. (RCT or Trial* or randomi* or random* allocat* or random* assign* or (control* adj1 Intervention*) or (treatment* adj1 control*) or evaluat* study or control group* or control condition* or comparison group* or comparison condition* or time series or (before adj1 after) or pre post or longitudinal or repeated measures or effect size* or comparative effective* or experiment* or pre-experiment* or difference in difference* or instrumental variable* or Propensity score or (control* adj1 treat*) or wait* list or quasi ex* or quasiexperiment* or matched control or matched comparison).ti.
17. (RCT or Trial* or randomi* or random* allocat* or random* assign* or (control* adj1 Intervention*) or (treatment* adj1 control*) or evaluat* study or control group* or control condition* or comparison group* or comparison condition* or time series or (before adj1 after) or pre post or longitudinal or repeated measures or effect size* or comparative effective* or experiment* or pre-experiment* or difference in difference* or instrumental variable* or propensity score or (control* adj1 treat*) or wait* list or quasi ex* or quasiexperiment* or matched control or matched comparison).ab
18. clinical trial/ or observational study/ or comparative study/ or evaluation study/
19. case-control studies/ or cohort studies/ or follow-up studies/ or longitudinal studies/ or prospective studies/ or retrospective studies/ or controlled before-after studies/ or cross-sectional studies/ or historically controlled study/ or interrupted time series analysis/ or feasibility studies/
20. 16 or 17 or 18 or 19
21. 15 and 20

EMBASE via Ovid

1. foster care/ or foster child/
2. child welfare/ or child protection
3. (foster adj2 (youth or child* or care)).ti.
4. (foster adj2 (youth or child* or care)).ab.
5. independent living/ or independent living program.mp.
6. independent living.ti.
7. independent living.ab.
8. (extend* adj2 (care or foster* or out of home care or OOHC or looked after)).mp.
9. (leav* adj2 (care or foster* or out of home care or OOHC or looked after)).mp.
10. (transit* adj2 (care or foster* or out of home care or OOHC or looked after)).mp.
11. (ag* out adj2 (care or foster* or out of home care or OOHC or looked after)).mp.
12. (emancipat* adj2 (care or foster* or out of home care or OOHC or looked after)).mp
13. self care/ or self care skills.mp.
14. 1 or 2 or 3 or 4
15. 5 or 6 or 7 or 8 or 9 or 10 or 11 or 12 or 13
16. 14 and 15
17. (RCT or Trial* or randomi* or random* allocat* or random* assign* or (control* adj1 Intervention*) or (treatment* adj1 control*) or evaluat* study or control group* or control condition* or comparison group* or comparison condition* or time series or (before adj1 after) or pre post or longitudinal or repeated measures or effect size* or comparative effective* or experiment* or pre-experiment* or difference in difference* or instrumental variable* or propensity score or (control* adj1 treat*) or wait* list or quasi ex* or quasiexperiment* or matched control or matched comparison).ti.
18. (RCT or Trial* or randomi* or random* allocat* or random* assign* or (control* adj1 Intervention*) or (treatment* adj1 control*) or evaluat* study or control group* or control condition* or comparison group* or comparison condition* or time series or (before adj1 after) or pre post or longitudinal or repeated measures or effect size* or comparative effective* or experiment* or pre-experiment* or difference in difference* or instrumental variable* or propensity score or (control* adj1 treat*) or wait* list or quasi ex* or quasiexperiment* or matched control or matched comparison).ab
19. clinical study/ or case control study/ or intervention study/ or longitudinal study/ or major clinical study/ or prospective study/ or retrospective study/ or comparative study/ or

controlled study/ or experimental study/ or feasibility study/ or observational study/ or quasi experimental study/ or replication study/ or cross-sectional study/ or controlled clinical trial/ or pretest posttest control group design/ or static group comparison/ or cross-sectional study/ or outcome assessment/
20. 17 or 18 or 19
21. 16 and 20

Sociological Abstracts via Proquest

1. SU.EXACT.EXPLODE(“Foster Children”) OR SU.EXACT(“Child Welfare Services”) OR SU.EXACT.EXPLODE(“Foster Care”) OR SU.EXACT(“Surrogate Parents”)
2. (ti(foster N/2 child*) OR ti(foster N/2 parent*) OR ti(foster N/2 care*) OR ti(foster N/2 home*)) OR (ab(foster N/2 child*) OR ab(foster N/2 parent*) OR ab(foster N/2 care*) OR ab(foster N/2 home*))
3. S1 OR S2
4. MAINSUBJECT.EXACT(“Self Care”) OR MAINSUBJECT.EXACT(“Deinstitutionalization”) OR MAINSUBJECT.EXACT.EXPLODE(“Independent Living”) OR MAINSUBJECT.EXACT(“Independence”)
5. (extend* NEAR/2 (care or foster*))
6. (leav* NEAR/2 (care OR foster*))
7. (transit* NEAR/2 (care OR foster*))
8. (ag* out NEAR/2 (care OR foster*))
9. S4 OR S5 OR S6 OR S7 OR S8
10. S3 AND S9
11. MAINSUBJECT.EXACT(“Empirical Methods”) OR MAINSUBJECT.EXACT(“Treatment”) OR MAINSUBJECT.EXACT(“Quantitative Methods”) OR MAINSUBJECT.EXACT(“Evaluation”) OR MAINSUBJECT.EXACT(“Statistical Significance”) OR MAINSUBJECT.EXACT(“Treatment Programs”) OR MAINSUBJECT.EXACT(“Placebo Effect”) OR MAINSUBJECT.EXACT(“Research Methodology”) OR MAINSUBJECT.EXACT(“Treatment Outcomes”) OR MAINSUBJECT.EXACT(“Effectiveness”) OR MAINSUBJECT.EXACT (“RANDOMNESS”)
12. (quasi-experimental OR quasi-experiment or quasiexperiment OR “propensity score” OR "control group*" OR “control condition*” OR "treatment group*" OR "comparison group*" OR "wait-list*" OR "waiting list*" OR "intervention group*" OR "experimental group*" OR "matched control" OR "matched group*" OR "matched comparison" OR "experimental trial" OR "experimental design" OR "experimental method*" OR "experimental stud*" OR "experimental evaluation" OR "experimental test*" OR "experimental assessment" OR "comparison sample" OR “propensity matched” OR “control sample” OR “control subject*” OR “intervention sample” OR “no treatment group” OR “nontreatment control” OR “pseudo experimental” OR “pseudo randomi?ed” OR “quasi-RCT” OR “quasi-randomi?ed” OR “compared with control*” OR “compared to control*” OR ”compared to a control*” OR “non-randomi?ed controlled stud*” OR “nonrandomly assigned”)
13. ti((RCT OR Trial* OR randomi* OR “random* allocat*” OR “random* assign*” OR (control* n/1 intervention*) OR (treatment* n/1 control*) OR “evaluat* study” OR “control group*” OR “control condition*” OR “comparison group*” OR “comparison condition*” OR “time series” OR “before after”) OR (“pre post” OR longitudinal OR “repeated measures” OR “effect size*” OR “comparative effective*” OR experiment* OR pre-experiment* OR “difference in difference*” OR “instrumental variable*” OR “propensity score” OR (control* n/1 treat*) OR “wait* list” OR “quasi ex*” OR quasiexperiment* OR “matched control” OR “matched comparison”))
14. ab((RCT OR Trial* OR randomi* OR “random* allocat*” OR “random* assign*” OR (control* n/1 intervention*) OR (treatment* n/1 control*) OR “evaluat* study OR “control group*” OR “control condition*” OR “comparison group*” OR “comparison condition*” OR “time series” OR “before after”) OR (“pre post” OR longitudinal OR “repeated measures” OR “effect size*” OR “comparative effective*” OR experiment* OR pre-experiment* OR “difference in difference*” OR “instrumental variable*” OR “propensity score” OR (control* n/1 treat*) OR

"wait* list" OR quasi ex* OR quasiexperiment* OR "matched control" OR "matched comparison"))
15. S11 OR S12 OR S13 OR S14
16. S10 AND S15

Social Services Abstracts via Proquest

1. SU.EXACT.EXPLODE("Foster Children") OR SU.EXACT("Child Welfare Services") OR SU.EXACT.EXPLODE("Foster Care") OR SU.EXACT("Surrogate Parents")
2. (ti(foster N/2 child*) OR ti(foster N/2 parent*) OR ti(foster N/2 care*) OR ti(foster N/2 home*)) OR (ab(foster N/2 child*) OR ab(foster N/2 parent*) OR ab(foster N/2 care*) OR ab(foster N/2 home*))
3. S1 OR S2
4. MAINSUBJECT.EXACT("Self Care") OR MAINSUBJECT.EXACT("Deinstitutionalization") OR MAINSUBJECT.EXACT.EXPLODE("Independent Living") OR MAINSUBJECT.EXACT("Independence")
5. (extend* NEAR/2 (care or foster*))
6. (leav* NEAR/2 (care OR foster*))
7. (transit* NEAR/2 (care OR foster*))
8. (ag* out NEAR/2 (care OR foster*))
9. S4 OR S5 OR S6 OR S7 OR S8
10. S3 AND S9
11. MAINSUBJECT.EXACT("Empirical Methods") OR MAINSUBJECT.EXACT("Treatment") OR MAINSUBJECT.EXACT("Quantitative Methods") OR MAINSUBJECT.EXACT("Evaluation") OR MAINSUBJECT.EXACT("Statistical Significance") OR MAINSUBJECT.EXACT("Treatment Programs") OR MAINSUBJECT.EXACT("Placebo Effect") OR MAINSUBJECT.EXACT("Research Methodology") OR MAINSUBJECT.EXACT("Treatment Outcomes") OR MAINSUBJECT.EXACT("Effectiveness") OR MAINSUBJECT.EXACT ("RANDOMNESS")
12. (quasi-experimental* OR quasi-experiment OR quasiexperiment OR "propensity score*" OR "control* group*" OR "control condition*" OR "treatment group*" OR "comparison group*" OR "wait-list*" OR "waiting list*" OR "intervention group*" OR "experimental group*" OR "matched control*" OR "matched groups" OR "matched comparison" OR "experimental trial" OR "experimental design" OR "experimental method*" OR "experimental stud*" OR "experimental evaluation" OR "experimental test*" OR "experimental assessment" OR "comparison sample" OR "propensity matched" OR "control sample" OR "control subject*" OR "intervention sample" OR "no treatment group" OR "nontreatment control" OR "pseudo experimental" OR "pseudo randomi?ed" OR quasi-RCT OR quasi-randomi?ed OR "compared with control*" OR "compared to control*" OR "compared to a control*" OR "non-randomi?ed controlled stud*" OR "nonrandomly assigned")
13. ti((RCT OR Trial* OR randomi* OR "random* allocat*" OR "random* assign*" OR (control* n/1 Intervention*) OR (treatment* n/1 control*) OR "evaluat* study" OR "control group*" OR "control condition*" OR "comparison group*" OR "comparison condition*" OR "time series" OR "before after") OR ("pre post" OR longitudinal OR "repeated measures" OR "effect size*" OR comparative effective* OR experiment* OR pre-experiment* OR "difference in difference*" OR "instrumental variable*" OR "propensity score" OR (control* n/1 treat*) OR "wait* list" OR "quasi ex*" or quasiexperiment* OR "matched control" OR "matched comparison"))
14. ab((RCT OR Trial* OR randomi* OR "random* allocat*" OR "random* assign*" OR (control* n/1 Intervention*) OR (treatment* n/1 control*) OR "evaluat* study" OR "control group*" OR "control condition*" OR "comparison group*" OR "comparison condition*" OR "time series" OR "before after") OR ("pre post" OR longitudinal OR repeated measures OR effect size* OR comparative effective* OR experiment* OR pre-experiment* OR "difference in difference*" OR "instrumental variable*" OR "propensity score" OR (control* n/1 treat*) OR "wait* list" OR "quasi ex*" or quasiexperiment* OR "matched control" OR "matched comparison"))
15. S11 OR S12 OR S13 OR S14
16. S10 AND S15

SocIndex via EBSCO

1. ((DE “FOSTER home care”) OR (DE “FOSTER mothers”) OR (DE “FOSTER parents”) OR (DE “FOSTER children”) OR (DE “FOSTER grandparents”) OR (DE “CHILD protection services”))
2. TI foster n2 child* OR TI foster n2 youth OR TI foster n2 parent* OR TI foster n2 care* OR TI foster n2 home OR TI “foster famil*” OR TI “fostering orphan*” OR TI “looked after children” OR TI “out of home care” OR TI “out of home placement” OR TI “substitute care” OR TI “looked after youth*”
3. AB foster n2 child* OR AB foster n2 youth OR AB foster n2 parent* OR AB foster n2 care* OR AB foster n2 home OR AB “foster famil*” OR AB “fostering orphan*” OR AB “looked after children” OR AB “out of home care” OR AB “out of home placement” OR AB “substitute care” OR AB “looked after youth*”
4. (extend* n2 (care or foster*))
5. (leav* n2 (care or foster*))
6. (transit* n2 (care or foster*))
7. (ag* out n2 (care or foster*))
8. DE "LIFE skills"
9. 1 or 2 or 3
10. 4 or 5 or 6 or 7 or 8
11. 9 and 10
12. DE “CLINICAL trials” OR DE “RANDOMIZED controlled trials” OR DE “OUTCOME assessment (Social services)” OR DE “SOCIAL services -- Evaluation” OR DE “FOLLOW-up studies (Medicine)” OR DE “PLACEBOS (Medicine)” OR DE “BLIND experiment” OR placebo* OR random* OR “comparative stud*” OR clinical NEAR/3 trial* OR research NEAR/3 design OR evaluat* NEAR/3 stud* OR prospectiv* NEAR/3 stud* OR (singl* OR doubl* OR trebl* OR tripl*) NEAR/3 (blind* OR mask*)
13. TI cohort* OR AB cohort* OR TI case-control* OR AB case-control* OR TI cross-section* OR AB cross-section* OR TI comparative* OR AB comparative* OR TI “validation stud*” OR AB “validation stud*” OR TI “evaluation stud*” OR AB “evaluation stud*” OR TI random* OR TI longitudinal* OR AB longitudinal* OR TI follow-up OR AB follow-up OR TI prospective OR AB prospective OR TI retrospective OR AB retrospective OR TI experimental OR AB experimental OR AB random*
14. (quasi-experimental OR quasi-experiment OR quasiexperiment* OR “propensity score*” OR ”control group*” OR “control condition*” OR “treatment group*” OR“wait-list*” OR “waiting list*”" OR “intervention group*” OR “experimental group*” OR “matched control” OR “matched groups” OR “matched comparison” OR “experimental trial” OR “experimental design” OR “experimental method*” OR “experimental stud*” OR “experimental evaluation” OR “experimental test*” OR “experimental assessment” OR "comparison sample" OR “propensity matched” OR “control sample” OR “control subject*” OR “intervention sample” OR “no treatment group” OR” nontreatment control” OR “pseudo experimental” OR “pseudo randomi?ed” OR “quasi-RCT” OR “quasi-randomi?ed” OR “compared with control*” OR “compared to control*” OR “compared to a control*” OR “non-randomized controlled stud*” OR “nonrandomly assigned”)
15. 12 or 13 or 14
16. 11 and 15

NHS Economic Evaluation Database via Ovid

1. Child welfare/
2. (foster adj2 (youth or child* or care)).mp.
3. independent living.ti
4. self Care/
5. (extend* adj2 (care or foster* or out of home care or OOHC or looked after)).mp.
6. (leav* adj2 (care or foster* or out of home care or OOHC or looked after)).mp.
7. (transit* adj2 (care or foster* or out of home care or OOHC or looked after)).mp.
8. (ag* out adj2 (care or foster* or out of home care or OOHC or looked after)).mp.
9. (empancipat* adj2 (care or foster* or out of home care or OOHC or looked after)).mp

10. 1 or 2
11. 3 or 4 or 5 or 6 or 7 or 8 or 9
12. 10 and 11

Health Technology Assessment via Ovid

1. Foster Home Care/
2. Child Welfare/
3. (foster adj2 (youth or child or care)).mp
4. independent living.mp
5. (extend* adj2 (care or foster* or out of home care or OOHC or looked after)).mp.
6. (leav* adj2 (care or foster* or out of home care or OOHC or looked after)).mp.
7. (transit* adj2 (care or foster* or out of home care or OOHC or looked after)).mp.
8. (ag* out adj2 (care or foster* or out of home care or OOHC or looked after)).mp.
9. (empancipat* adj2 (care or foster* or out of home care or OOHC or looked after)).mp
10. self care/
11. 1 or 2 or 3
12. 4 or 5 or 6 or 7 or 8 or 9 or 10
13. 11 and 12
